# Diabetes and Osteoporosis – Cause for Concern?

**DOI:** 10.3389/fendo.2014.00053

**Published:** 2014-04-16

**Authors:** Peter Vestergaard

**Affiliations:** ^1^Department of Clinical Medicine, Aalborg University Hospital, Aalborg, Denmark; ^2^Department of Endocrinology, Aalborg University Hospital, Aalborg, Denmark

**Keywords:** diabetes, osteoporosis, bone quality, bone, endocrinology and bone metabolism

Diabetes and osteoporosis are both frequent disorders. By chance they may thus occur together. However, evidence suggests that osteoporosis defined by bone scans (DXA) occurs more frequent than expected in type 1 diabetes (T1D) and less frequent than expected in T2D (1). The latter may be explained by differences in body size – T2D patients being more obese than the general population and bone mineral density (BMD) increases with body size (2). However, as disclosed by prior studies and by the papers in this research topic, more is present than what meets the eye at first glance on the bone density measures. More fractures are present than expected both in T1D and T2D. This could point at either a diminished bone biomechanical competence or an increased risk of trauma. The latter seems less likely. Regarding bone biomechanical competence, this series of papers has shown discordances in biochemical markers of bone turnover (3). This raises the question of the potential mechanisms of uncoupling of formation and resorption, and thus a potential weakening of bone microarchitecture. Besides the issue of the interactions with bone cells such as the osteoclasts, osteocytes, and osteoblasts, interactions with both the calcium matrix and thus the crystalline structure of hydroxyapatite and interactions with collagen are possible. This opens for a wide range of possible research fields within signaling [bone – adipose tissue (4), bone – pancreas etc.], imaging (crystal structure), engineering (biomechanical properties) etc.

Within each of these fields, a number of questions need to be addressed. Are the measurements of biochemical markers of bone turnover reliable? One example could be if enzymatic or non-enzymatic modification of say collagen cross-links could infer with the accuracy of assays, thus measuring lower concentrations as not all of the degraded collagen is detected. Furthermore, many interactions between glucose and osteocalcin (5) modify the effectiveness of this as a marker of bone turnover.

The change in biochemical markers happen rather rapidly and were in one study modified by somatostatin. The rapid changes raise novel questions as bone turnover usually is a rather slow process that takes more than hours to modify substantially. However, the interaction with somatostatin raises the possibility of an interaction from gut hormones (6) and ghrelin. Studies of a broader range of hormonal signaling may thus be in place.

The field of biomechanical competence may call for novel imagining techniques and new methods to study crystal structure and biomechanical competence.

The study of diabetes and bone thus not only relates to classical endocrinology and bone metabolism, but also opens new concepts of bone biomechanical competence beyond traditional DXA techniques and even beyond HrpQCT and beyond classical biochemical markers of bone turnover.

## Conflict of Interest Statement

The author declares that the research was conducted in the absence of any commercial or financial relationships that could be construed as a potential conflict of interest.
